# Case report: Acute hepatic failure secondary to metastatic LIVER’S infiltration by upper tract urothelial carcinoma

**DOI:** 10.1016/j.amsu.2019.07.019

**Published:** 2019-07-11

**Authors:** Francesco Serra, Cristiano Guidetti, Francesco Spatafora, Francesca Cabry, Alberto Farinetti, Vittoria Mattioli Anna, Roberta Gelmini

**Affiliations:** Department of Surgery, University of Modena and Reggio Emilia – Policlinico of Modena, Modena Italy Via del Pozzo, 71 41100, Modena, Italy

**Keywords:** Acute liver failure, Upper tract urothelial carcinoma (UTUC), Emergency surgery

## Abstract

**Introduction:**

Acute liver failure (ALF) secondary to malignant infiltration of the liver from urothelial carcinoma is a very rare clinical condition and is often diagnosed only after death. Upper tract urothelial carcinoma (UTUC) is a rare, from 5% to 10% of all urothelial tumours, but possible cause of ALF when there is extensive liver metastatic involvement. We report the case of a patient who died in the intensive care unit (ICU) of our hospital from multiple organ failure (MOF) secondary to ALF, as a result of infiltration of the liver from UTUC diagnosed after surgery.

PRESENTATION OF THE CASE: A 69-year-old Caucasian man was referred to our hospital for hematuria, melena, right upper quadrant (RUQ) pain and jaundice developed over the previous two weeks. After multidisciplinary discussion, he underwent emergency exploratory laparotomy to perform cholecystectomy because of suspected acute cholecystitis considered as a septic focus within the left kidney. He developed MOF and died on the 6th postoperative day.

**Discussion:**

From the diagnosis of the renal mass and the death of the patient, a few days have passed, and the diagnosis of UTUC has been put only at histological examination.The most common sites of metastases from UTUC are lymph nodes, lungs, liver, bones and peritoneum. Moreover, liver metastases have been identified to have an independent negative impact on overall survival in a patient affected by UTUC.

**Conclusion:**

The authors suggest that this condition should be taken into account when dealing with patients with evidence of a renal mass and simultaneous ALF.

## Highlights

• Unusual clinical presentation of acute liver failure from upper urothelial carcinoma (UTUC).
• Rarely these diseases are managed in the emergency setting.
• This condition should be taken into account when dealing with patients with evidence of a renal mass and simultaneous ALF.

The work was written in line with the SCARE criteria [1] Consent to the processing of data for scientific purposes and for possibile pubblication is requested to patient and signed at the time of admission and kept in the medical record.

## 1 Introduction

Acute liver failure (ALF) secondary to malignant infiltration of the liver from urothelial carcinoma is a very rare clinical condition and is often diagnosed only after death [2]. Upper tract urothelial carcinoma (UTUC) is a rare, from 5% to 10% of all urothelial tumours [3], but possible cause of ALF when there is extensive liver metastatic involvement [4]. Rarely these disease are managed in the emergency setting, because are often diagnosed by urologists and the overall survival is about 34 months for the UTUC itself; the most recognized risk factors that cause this tumous are cigarette smoking, exposure to chemical solvents, exposure to arsenic, the use of phenacethine; there is also a clinical entity, the Balkan endemic nephropathy, which determines an interstitial inflammatory damage responsible for 50% of renal tumours in that region [4].

We report the case of a patient who died in the intensive care unit (ICU) of our hospital from multiple organ failure (MOF) secondary to ALF, as a result of infiltration of the liver from UTUC diagnosed after surgery The rate of systemic metastasis in UTUC with muscle invasion is about 30% [5]. Only a minority of patients with this condition present involvement of extra-regional lymph nodes and other distant metastasis at the moment of diagnosis [6].

## 2 Presentation of the Case

A 69-year-old Caucasian man was referred to our hospital for hematuria, melena, right upper quadrant (RUQ) pain and jaundice developed over the previous two weeks. In his past medical history, the only cholelithiasis was recorded. Physical examination highlighted melena, hemoptysis and tenderness in the RUQ. Laboratory tests run in the emergency department revealed a level of hemoglobin of 7g/dl, platelets 39.000 Migl/mmc, white blood cell 13.99 Migl/mmc (neutrophils 69.9%), PT 1.61, aPTT 3.10, albumin 2.2 g/dl, total bilirubin 9.23 mg/dl, direct bilirubin 7.53 mg/dl, aspartate aminotransferase (AST) 213 IU/l, and alkaline phosphatase 567 IU/l, Creatinine 1.32mg/dl, CRP 5.5 mg/dl.

**Fig. 1:**
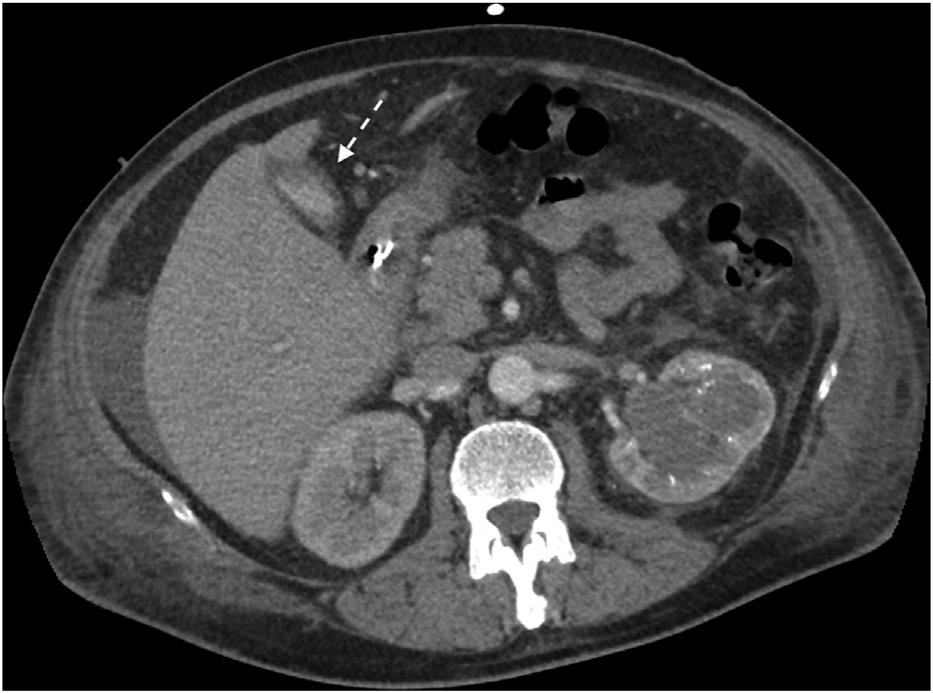
Pre-operative TC that highlight the presence of a thickened wall of the gallbladder.

**Fig. 2:**
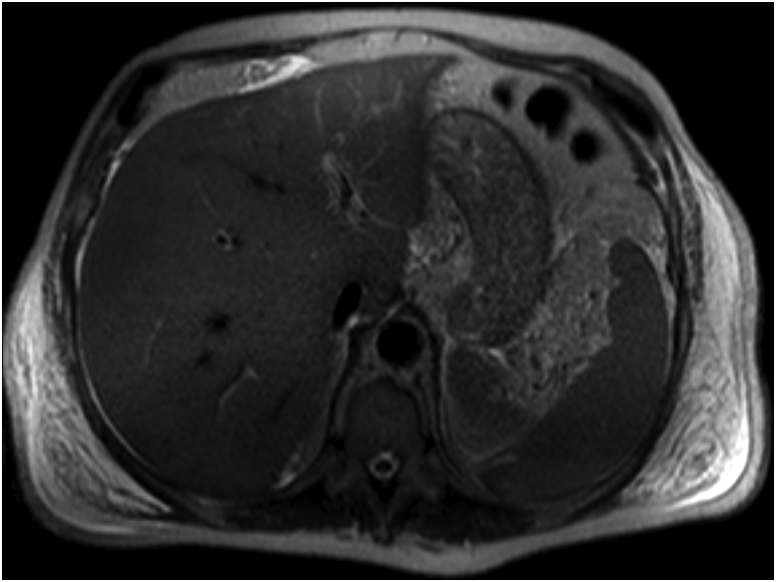
RM: no signs of hepatic infiltration or metastasis.

CT scan of the chest and the abdomen was performed in the emergency department. Diffuse alveolar haemorrhage with right pleural effusion and mediastinal lymphadenopathy was demonstrated. In the abdomen, the main finding was a large solid lesion of the left kidney (6.5 cm × 6.2 cm), with multiple regional lymphadenopathies and a homogeneously enlarged liver without evident focal lesions. The gallbladder had focal thickening of its walls. Also, the RMI confirmed the presence of a thickened wall of the gallbladder with a surrounding thin fluid layer; no signs of hepatic infiltration from solid organ tumour was detected (Fig. 1, Fig. 2).

The patient was brought to the ICU and required ventilatory support. It was necessary to transfuse him with multiple packed red blood cells, plasma and platelets to revert the hemorrhagic shock he developed soon after hospitalisation, due to bleeding from the left kidney's mass. He also underwent left renal artery embolisation to control the urinary tract bleeding. Since LFTs were out of range, hepatitis A, B, C and E serologies were tested in the suspect of acute hepatitis. They all came back negative. The patient had no history of alcohol abuse, and there were no signs of hepatotoxic medications intake.

After multidisciplinary discussion, he underwent emergency exploratory laparotomy to perform cholecystectomy because of suspected acute cholecystitis considered as a septic focus within the left kidney. At the time of the laparotomy, the liver appeared yellowish with thin fibrous streaks, no signs of acute cholecystitis were observed. However, the cholecystectomy was performed, such as a left nephrectomy and a wide liver biopsy. After the surgical procedure, the clinical condition of the patient rapidly worsened: he developed atrial fibrillation, acute renal failure and respiratory insufficiency. He developed MOF and died on the 6th postoperative day.

The result of histological exams confirmed multiple spots of high-grade carcinoma in hepatic tissue with an immunophenotype compatible with urothelial carcinoma (CK 5/6-; CK20+; CDX2-; PAX8-; TTF1-). A high-grade urothelial carcinoma (pT3) was detected in the left kidney. Gallbladder showed modest acute on chronic inflammation.

The autopsy revealed a grossly enlarged liver, that was granular in appearance with ‘‘salt and pepper’’ distribution of the metastases without any focal lesion. Microscopically there was the near-complete parenchymal replacement by metastatic tissue.

## 3 Discussion

We reported the clinical case of a patient who died in an intensive care unit where he was admitted directly from the emergency room in good general conditions but with an initial septic state. From the diagnosis of the renal mass and the death of the patient, a few days have passed, and the diagnosis of UTUC has been put only at histological examination. The absence of a diagnosis and the septic condition of the patient with a radiological doubt of cholecystitis as well as the need to remove the embolized kidney led the multidisciplinary team to decide for surgery. In literature we don't have found one case report similar to this; we have found that UTUC is uncommon and accounts for approximately 5% of all urothelial malignancies [3,7]. The treatment of choice for patients detected in advanced stage of the disease is the combination of cisplatin-based systemic chemotherapy and radical nephroureterectomy [8]. UTUC also had a high potential for local and distant recurrence [9]. The most common sites of metastases from UTUC are lymph nodes, lungs, liver, bones and peritoneum. Moreover, liver metastases have been identified to have an independent negative impact on overall survival in a patient affected by UTUC [10]. Some author had described that in elderly patients with poor possibilities of survival, the watchful-waiting approach, without surgery, may extend the survival [11].

Furthermore liver metastases from UTUC are usually multifocal hypodense lesions. In our case the immunophenotype found on liver biopsy specimen evidenced cytokeratin 20 expression associated with low expression of cytokeratin 5/6, that is typical for poorly differentiated tumours characterised by high invasiveness [12]. In literature, the most frequent cause of ALF caused by metastatic infiltration of cancer is another kind of cancer such as small cell lung cancer or neuroendocrine carcinomas and the differential diagnosis has to be done mainly with sepsis and paraneoplastic syndromes such as Stauffer syndrome [13]. Laboratory findings highlighted several negative prognostic serum markers for high-grade UTUC, such as severe anaemia, low levels of albumin and high level of alkaline phosphatase that are described in the literature to be related with advanced pathologic T stage, not an organ-confined disease, lymph-vascular invasion, and tumour necrosis [14].

## 4 Conclusion

We have described a very rare case that has involved many specialists in emergency department characterised by a challenging clinical condition that had been treated with surgery in an extreme attempt to save the patient. With this case report, the authors suggest that this condition should be taken into account when dealing with patients with evidence of a renal mass and simultaneous ALF.

## Ethical approval

No ethical approval was required and consent to the processing of data for scientific purposes and for possibile pubblication is requested to patient and signed at the time of admission and kept in the medical record.

## Sources of funding

Non funding were used.

## Author contribution

Authors:

Serra Francesco, MD.

Department of Surgery, University of Modena and Reggio Emilia – Policlinico of Modena, Modena Italy.

Via del Pozzo, 71 41100 Modena Tel: +390594223662 FAX: +390594224370.

serrafrancescomd@gmail.com.

Author and supervisor of entire manuscript.

Guidetti Cristiano, MD.

Department of Surgery, University of Modena and Reggio Emilia – Policlinico of Modena, Modena Italy.

Via del Pozzo, 71 41100 Modena Tel: +390594223662 FAX: +390594224370.

cristianogui@yahoo.it.

Co-author of entire manuscript.

Spatafora Francesco, MD.

Department of Surgery, University of Modena and Reggio Emilia – Policlinico of Modena, Modena Italy.

Via del Pozzo, 71 41100 Modena Tel: +390594223662 FAX: +390594224370.

Spataf86@virgilio.it.

Data collection and co-author of case report and discussion.

Cabry Francesca, MD.

Department of Surgery, University of Modena and Reggio Emilia – Policlinico of Modena, Modena Italy.

Via del Pozzo, 71 41100 Modena Tel: +390594223662 FAX: +390594224370.

francesca.cabry@unimore.it.

Data and imaging collection.

Farinetti Alberto, MD.

Department of Surgery, University of Modena and Reggio Emilia – Policlinico of Modena, Modena Italy.

Via del Pozzo, 71 41100 Modena Tel: +390594223662 FAX: +390594224370.

alberto.farinetti@unimore.it.

Review of literature.

Mattioli Anna Vittoria, MD.

Department of Surgery, University of Modena and Reggio Emilia – Policlinico of Modena, Modena Italy.

Via del Pozzo, 71 41100 Modena Tel: +390594223662 FAX: +390594224370.

annavittoria.mattioli@unimore.it.

Review of literature and co-author of discussion.

Gelmini Roberta, MD PhD.

Department of Surgery, University of Modena and Reggio Emilia – Policlinico of Modena, Modena, Italy.

Via del Pozzo, 71 41100 Modena Tel: +390594223662 FAX: +390594224370.

roberta.gelmini@unimore.it.

Supervisor and co-author of entire manuscript.

## Conflicts of interest

No conflicts of interest.

## Trial registry number – ISRCTN

None.

## Guarantor

Gelmini Roberta, MD PhD.

Dept. of Surgery – Policlinico of Modena, University of Modena and Reggio Emilia –

Via del Pozzo, 71 41124 Modena, Italy.

Tel: +39 0594223662.

FAX: +39 0594224370.

e_mail: roberta.gelmini@unimore.it.

## Research registration unique identifying number (UIN)

The submitted case report is not a research study.

## Patient consent

Consent to the processing of data for scientific purposes and for possibile pubblication is requested to patient and signed at the time of admission and kept in the medical record.

## Provenance and peer review

Not commissioned, externally peer reviewed.

## Disclosure statement

The authors have nothing to disclose.
